# The Value of Bi-Chloride of Mercury in the Treatment of Urethritis

**Published:** 1892-02

**Authors:** 


### THE VALUE OF BI-CHLORIDE OF MERCURY IN THE TREATMENT OF URETHRITIS.

BREWER (*International Journal of Surgery*, November, 1881) reiterates his confidence in the efficacy of bi-chloride of mercury in the treatment of urethritis. His method was as follows: At the first visit the patient was instructed in the proper use of a syringe, and was given a large amount of a solution of bi-chloride of mercury, varying in strength from 1: 16,000 to 1: 50,000, according to the sensitiveness of his urethra, and the stage of the disease. This he was instructed to use twice daily, by taking ten injections in the morning, and ten at night, holding each one in the uretha one minute to imitate as nearly as possible the result of irrigation. The patient was seen three times a week. As soon as the discharge lost its purulent character, bi-chloride was suspended and a mild astringent was substituted, preferably bismuth suspended in water. In the fifty five cases treated in this way, five were not benefitted, after an average employment of the method for seven days. In the remaining fifty cases, the average length of time necessary to effect a change in the discharge from pus to a thin watery secretion was a fraction over eight days. The discharge entirely disappeared on an average of twenty-one days. Epididymitis occurred in three of these cases, and posterior urethritis was developed in two. The reporter very fairly states that these statistics are of little value from a scientific point of view, since the cessation of the discharge can by no means be considered as an index that the disease has been cured. He offers his conclusions not so much on the basis of these cases, as upon a very large personal experience, and states positively that the judicious use of bi-chloride of mercury in cases of acute gonorrhœal urethritis is attended with better results in subduing the painful and disagreeable features of the disease, than is any other agent. The recovery is more rapid and permanent, and the frequency of inflammatory complications is greatly reduced.— *The Therapeutic Gazette.*

